# CRIM1 Complexes with ß-catenin and Cadherins, Stabilizes Cell-Cell Junctions and Is Critical for Neural Morphogenesis

**DOI:** 10.1371/journal.pone.0032635

**Published:** 2012-03-12

**Authors:** Virgilio G. Ponferrada, Jieqing Fan, Jefferson E. Vallance, Shengyong Hu, Aygun Mamedova, Scott A. Rankin, Matthew Kofron, Aaron M. Zorn, Rashmi S. Hegde, Richard A. Lang

**Affiliations:** 1 The Visual Systems Group, Cincinnati Children's Hospital Medical Center, University of Cincinnati, Cincinnati, Ohio, United States of America; 2 Divisions of Pediatric Ophthalmology, Cincinnati Children's Hospital Medical Center, University of Cincinnati, Cincinnati, Ohio, United States of America; 3 Divisions of Developmental Biology, Cincinnati Children's Hospital Medical Center, University of Cincinnati, Cincinnati, Ohio, United States of America; 4 Department of Ophthalmology, University of Cincinnati, Cincinnati, Ohio, United States of America; 5 Molecular and Developmental Biology Graduate Program, University of Cincinnati, Cincinnati, Ohio, United States of America; University of Colorado Boulder, United States of America

## Abstract

In multicellular organisms, morphogenesis is a highly coordinated process that requires dynamically regulated adhesion between cells. An excellent example of cellular morphogenesis is the formation of the neural tube from the flattened epithelium of the neural plate. Cysteine-rich motor neuron protein 1 (CRIM1) is a single-pass (type 1) transmembrane protein that is expressed in neural structures beginning at the neural plate stage. In the frog *Xenopus laevis*, loss of function studies using CRIM1 antisense morpholino oligonucleotides resulted in a failure of neural development. The CRIM1 knockdown phenotype was, in some cases, mild and resulted in perturbed neural fold morphogenesis. In severely affected embryos there was a dramatic failure of cell adhesion in the neural plate and complete absence of neural structures subsequently. Investigation of the mechanism of CRIM1 function revealed that it can form complexes with ß-catenin and cadherins, albeit indirectly, via the cytosolic domain. Consistent with this, CRIM1 knockdown resulted in diminished levels of cadherins and ß-catenin in junctional complexes in the neural plate. We conclude that CRIM1 is critical for cell-cell adhesion during neural development because it is required for the function of cadherin-dependent junctions.

## Introduction

Development of multicellular organisms requires the coordinated movement of cells in a process generally referred to as morphogenesis. Morphogenesis at the organismal scale can be dramatic – for example, the closure of the neural tube – and complex because it requires synchronizing distinct activities in multiple tissue layers. Component activities of morphogenesis include cell migration, cell elongation, process formation, coordinated shape change during epithelial bending as well as regionally increasing and decreasing tissue volumes driven by cell proliferation and cell death.

The regulation of adhesive interactions is a key factor in control of morphogenesis. Among adhesion molecules, cadherins have a critical role [1], [2], [3]. The classical cadherins exist in a complex with catenins. The catenins regulate association of cadherins with the actin cytoskeleton, though binding may not be direct [3], [4]. Association of cadherins with actin is likely mediated by the bridging molecule eplin [5]. Adhesive activity of cadherins can be regulated in a variety of ways [3] and this is clearly important in permitting and mediating the cellular movements of morphogenesis. In some settings, catenins are essential for cell-cell adhesion. For example, p120 catenin loss-of-function in the salivary gland results in severe defects in adhesion accompanied by the down-regulation of E-cadherin [6]. ß-catenin loss-of-function in the presumptive lens results in a reduction of the F-actin cytoskeleton and loss of cell adhesion [7]. Antisense oligonucleotide depletion of both α-catenin and EP-cadherin in *Xenopus* embryos causes a failure of cellular adhesion at blastula stages [8], [9]. A two-tiered regulation of E-cadherin has recently been reported in embryonic epithelia of *Drosophila* whereby a stable cell-cell homophillic E-cadherin complex pool and a more diffusible monomeric E-cadherin pool co-exist at cell junctions [10]. These pools of E-cadherin have different connections to the intracellular actin network and must require different mechanisms for turnover and regulation during embryonic morphogenesis.

Cysteine-rich motor neuron 1 (CRIM1) was originally identified as a partial cDNA in an interaction screen [11] and in a screen for secreted proteins (C. Tabin, personal communication). Assembly of the full sequence representing the *CRIM1* cDNA [11] revealed that it was a type 1 trans-membrane protein with N-terminal homology to insulin-like growth factor binding domains (IGFBP; [11], [12]) and a set of six cysteine-rich von Willebrand factor C (vWC) repeats occupying the remaining extracellular domain. The cysteine-rich repeats of CRIM1 are similar to those of chordin [13] and its *Drosophila* homolog, short gastrulation [14] that can bind bone morphogenetic proteins (BMPs) [15], [16]. Another protein that contains an IGFBP and single cysteine-rich domain is Cyr61, a secreted heparin binding, extracellular matrix associated protein that is required for normal gastrulation movements [17]. CRIM1 is expressed in a variety of tissues and cell types that include the vertebrate CNS [11] urogenital tract [18] eye [19], [20] and vascular system [21]. CRIM1 protein has been localized to the endoplasmic reticulum [21], [22] or to junctional complexes upon stimulation of vascular endothelial cells [21].

Analysis of CRIM1 function suggested it has a role in vascular tube formation both in culture [21] and in vivo in the fish [23]. Consistent with expression of CRIM1 in the neural tube [11], over-expression of the CRIM1 ectodomain in the chick neural tube reduces the numbers of certain spinal cord neurons [20]. CRIM1 was also been proposed to be an antagonist for bone morphogenetic proteins (BMPs) through suppression of BMP maturation and sequestration in the Golgi or at the cell surface [22]. This activity is dependent upon the extracellular vWC repeats [22]. Expression of CRIM1 in the chick neural tube was, however, insufficient to modulate ventral patterning [20] where BMP activity is critical [24]. An assessment of the function of *crm-1*, a *C. elegans* homologue of CRIM1, has suggested a role in enhancing BMP signaling [25]. Identification of a CRIM1 hypomorphic mutant in the mouse (*CRIM1^KST264^*, [26]) that was generated by lacz insertional mutagenesis has revealed that CRIM1 is involved in the development of multiple organ systems including the limbs, eye and kidney vascular system [26], [27].

In the current study we have focused on understanding the activity of the CRIM1 cytoplasmic domain, a region of 82 amino acids that is highly conserved. Antisense oligonucleotide mediated loss of function studies in *Xenopus laevis* revealed an essential role for CRIM1 in neural plate cell adhesion. In these experiments there was a loss of junctional cadherin labeling intensity, reduced epithelial polarity and organization and ultimately, the sloughing of neural plate cells. Based on this result we screened CRIM1 containing complexes for the presence of known adhesion mediators. We found that the cytoplasmic domain of CRIM1 can form complexes with ß-catenin and cadherins, though this interaction is probably indirect. Combined, these data suggest that CRIM1 is essential for cadherin mediated cell-cell adhesion in the developing nervous system.

## Materials and Methods

### Ethics Statements

All experiments were performed in accordance with institutional guidelines under Institutional Animal Care and Use Committee (IACUC) approval at Cincinnati Children's Hospital Research Foundation (CCHRF). IACUC at CCHRF approved the study described in this manuscript with Animal Use Protocol number 0B12097.

### Plasmid constructs

Plasmid constructs were generated using conventional methods using full-length CRIM1 cDNA *Xenopus laevis* EST 5537401 (Invitrogen).

### Cell lines and transfection

HEK 293T cells (ATCC, CRL-11268) were cultured in a conventional manner. Cell lines were transfected with DNA constructs using Fu-Gene (Roche) or Trans-IT (Mirus) reagents.

### Morpholino experiments and *in situ* hybridization

Translation-blocking morpholino oligonucleotides (MOs) were designed against xCRIM1a (XLCA) and xCRIM1b (XLCB) (Gene Tools, LLC). The MOs were prepared at a concentration of 30 mg/mL in sterile water. We used a 10,000 MW fluorescent dextran (Molecular Probes) or a GFP-encoding mRNA as lineage tracers.

*X. laevis* eggs were fertilized *in vitro* and grown in 0.1X modified Barth saline (MBS) [28], staged according to [29] and transferred to 1X MBS, 4% Ficoll for microinjection. Embryos were injected at the 4 to 16-cell stage in individual blastomeres and cultured in 0.1X MBS, 2% Ficoll at 18°C or in 0.1X MBS for longer incubations. Embryos were fixed in 1X MEMFA at various stages for analysis.

Antisense RNA probe synthesis and *in situ* hybridization on whole embryos were performed as previously described [30].

cDNA from staged *X. laevis* embryos (stages 8 to 32) were a generous gift from C. Wylie. We used PCR primers specific to the 5′-UTR of xCRIM to amplify sequences from isolated cDNAs.

### Protein analysis

Immunoblotting was performed using conventional chemiluminescence methods. For immunoprecipitation, we lysed the cells in a modifed IP buffer (50 mM Tris-Hcl, pH 7.4, 150 mM NaCl, 1 mM CaCl_2_, 1 mM MgCl_2_, 2% glycerol, 0.1% BSA, 0.5% NP-40, and 0.5% Triton X-100). Cell lysates were incubated with the antibodies and proteins precipitated with protein A agarose beads (Invitrogen). Antibodies used for immunoprecipitation and/or western analysis are as follows: mouse anti-V5 mAb (Invitrogen), mouse anti-FLAG mAb (Sigma), rabbit anti-ß-catenin (H-102, Santa Cruz), and mouse anti-N-cadherin (Zymed and H-63, Santa Cruz).

### Immunofluorescence labeling

Cryosection labeling was carried out as described [7] with rabbit anti-ß-catenin (1∶2500, Santa Cruz, SC-7199) and goat anti-rabbit Alexa568 secondary (1∶1000, Molecular Probes #A-11011) antibodies. Whole mount staining was carried out as previously described [31]. Antibodies were anti-C-cadherin or anti-E-cadherin (6B6 or 5D3, respectively; Developmental Study Hybridoma Bank, Iowa, 1∶250), anti-activated caspase-3 (1∶250, BD Pharmingen 559565) and rabbit-anti-GFP Alexa488 (1∶300, Molecular Probes). Secondary antibodies were Cy5 goat anti-mouse or Cy3 goat anti-rabbit IgG (1∶300, Jackson Laboratory). TUNEL labeling (Roche) was performed with manufacturer protocols. One-way ANOVA statistical analysis was performed using SPSS software.

## Results

### Knockdown of *CRIM1* in *Xenopus* embryos causes defects in neuronal structures development

**Figure 1:**
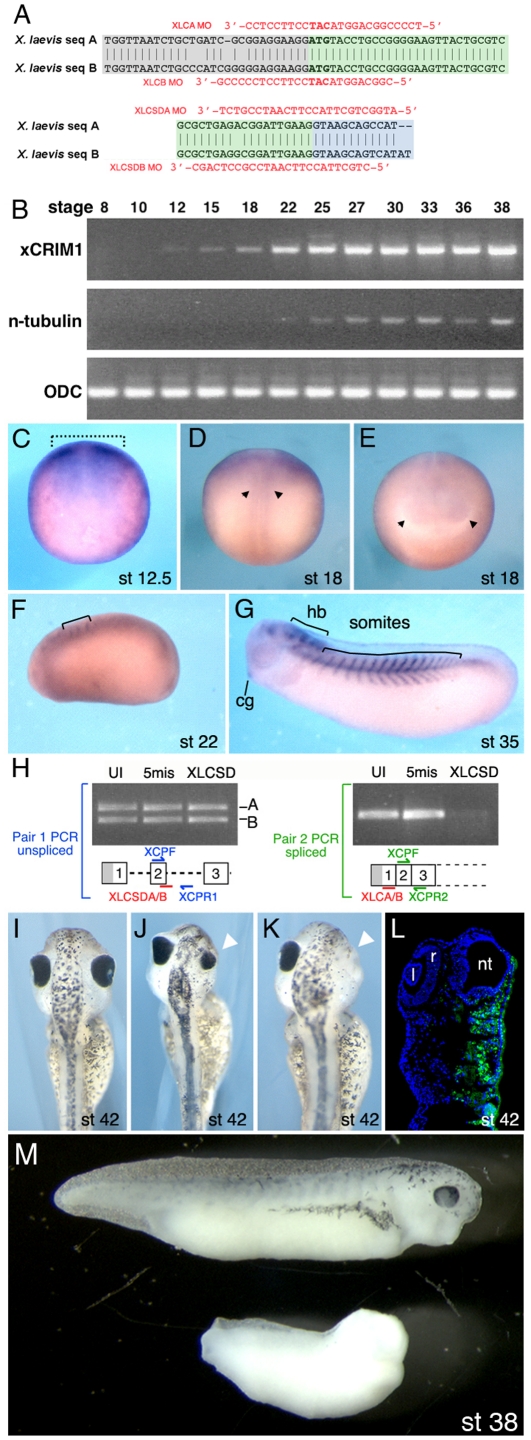
CRIM1 is expressed in the neural plate and is required for development of neural structures. (**A**) Antisense morpholino oligo (MO) sequences and their complimentary targets on the *Xenopus laevis* messenger sequences are indicated for both the translation blocking MOs (upper) and the splice-donor blocking MOs (lower). (**B**) RT-PCR assessment of *CRIM1A* transcript expression in developmentally staged *Xenopus laevis* embryos. *n-tubulin* is a neuronal differentiation marker and *ornithine decarboxylase* (*ODC*) a ubiquitously expressed control. (**C–G**) *In situ* hybridizations for *CRIM1A* in *Xenopus laevis* embryos. The dashed bracket in (C) indicates the neural plate region, the arrowheads in (D) the neural tube and in (E) the optic vesicles. cg: cement gland; hb: hindbrain. (**H**) Detection of unspliced or spliced *CRIM1A* and *B* mRNA in uninjected embryos (UI) or those injected with the 5-missense control (5mis) or splice blocking (XLCSD) MOs. The PCR primer pair XCPF and XCPR1 detects unspliced mRNA while XCPF and XCPR2 detect the spliced product. (**I–L**) *CRIM1* loss-of-function experiments where (I) is an uninjected control and (J–L) were injected with XLCA and B MOs at 15 ng each. Small (J, arrowhead) or missing eyes (K, arrowhead) often result. The embryo in (L) was coinjected with 10 kDa Alexa-fluor 488 dextran (green). l: lens; r: retina; nt: neural tube. (**M**) Comparison of control embryo (upper) and embryo injected at the 2-cell stage bi-laterally with XLCAB at 30 ng each (lower). Developmental stages as indicated.

Using available chick *CRIM1* sequence (accession #NM_204425) to design primer sets, we PCR amplified cDNA products from a stage 28 *Xenopus laevis* cDNA library and identified two distinct sequences that had extensive homology to chick *CRIM1*. Based on the high degree of homology, these clone families represented the *Xenopus laevis* A and B genes. We used this sequence information (Fig. 1A, accession number pending) to design PCR primers, antisense Morpholino oligonucleotides (MO, Fig. 1A, Table 1) and in situ hybridization probes for *Xenopus laevis CRIM1*.

**Table 1:** Morpholino oligonucleotides used in this study.

*X. laevis CRIM1* Morpholino Oligonucleotide Sequences:	
XLCA	5′-TCCCCGGCAGGTA**CAT**CCTTCCTCC-3′
XLCB	5′-CGGCAGGTA**CAT**CCTTCCTCCCCCG-3′
5MXLCA	5′-TCgCCcGCAGcTA**CAT**CgTTCgTCC-3′
5MXLCB	5′-CGcCAcGTA**CAT**TgTTCCTgCCgCG-3′
SCMO	5′-CCTCTTACCTCAGTTACAATTTATA -3′
XLCSDA	5′-ATGGCTGCTTACCTTCAATCCGTCT-3′
XLCSDB	5′-CTGCTTACCTTCAATCCGCCTCAGC-3′
5MXLCSDA	5′-ATcGCTcCTTAgCTTgAATCCcTCT-3′

Nucleotides complementary to the start codon of *CRIM1* are indicated in bold. Control Morpholinos have either a 5 nucleotide mismatch (5 M) or are the standard control Morpholino (SCMO) from GeneTools. Mismatched nucleotides are indicated in lower case.

RT-PCR analysis for *CRIM1* on a staged series of embryos (Fig. 1B) showed that *CRIM1* mRNA is detected in the early neurula at stage 12. For comparison, *n-tubulin* mRNA was detected in the late neurula at stage 22. By in situ hybridization, *CRIM1* was detected in the neural plate of stage 12.5 embryos (Fig. 1C). Expression of *CRIM1* in neural structures continued and at stage 18, albeit faintly detected, in posterior neural tube (Fig. 1D) as well as anterior neural structures including optic vesicles (Fig. 1E). *CRIM1* expression at stage 22 was detected in the early somites and weakly in neural structures (Fig. 1F). The hindbrain, cement gland and somites were all locations of *CRIM1* expression at stage 35 (Fig. 1G).

CRIM1 loss-of-function experiments in *Xenopus laevis* were performed using antisense MO-mediated translation and splicing blocking [32], [33]. Sequence differences in the 5′ untranslated region of the *CRIM1* A and B genes (Fig. 1A) required that we use a mixture of MOs (XLCAB, Table 1) for translation blocking. To design splicing-blocking MOs, we first identified *Xenopus tropicalis* genomic *CRIM1* sequences in the available database (JGI Genome Browser) and used that sequence to PCR amplify and sequence *Xenopus laevis* genomic clones. The *CRIM1* A and B genes also had sequence changes in the exon 2 splice donor region (Fig. 1A) that necessitated a mix of MOs (XLCSDAB, Table 1) to target both mRNAs. Using two sets of PCR primers that detected either unspliced or spliced mRNA (Fig. 1H) we confirmed that the MOs targeted to the splice donor of *CRIM1* exon 2 suppressed splicing.

Translation and splicing blocking CRIM1 MOs injected into a dorsal blastomere at the 4-cell stage produced dramatic effects on the development of neural structures. In a typical experiment where 15 ng each of XLCA and B were injected, more than 70% of embryos had major defects including a small or missing eye on the injected side (Table 2 and Fig. 1I, J, K, L). Tracing of MO distribution with coinjected Dextran Alexa488 confirmed that the affected region of the embryo received MO but that any remaining neural tube was tracer negative (Fig. 1L). Histological assessment of affected *Xenopus* embryos at stage 42 confirmed the neural tube and eye were both missing on the injected side (data not shown). In embryos injected bi-laterally at the 2-cell stage with 30 ng each XLCA and B MOs, a loss of anterior neural and head structures resulted but ventral and posterior structures were retained (Fig. 1M). This phenotype induced by loss of CRIM1 in the whole embryo by administering the MOs at this stage correlates well given the expression pattern of *CRIM1* in the developing neural plate.

**Table 2:** Phenotype summary for *CRIM1* Morpholino injections.

Morpholino Oligos Injected	Eye (Injected Side)			Total Embryos
	Normal	Small	Absent	
**Translation Blocking**				
Uninjected	100%	0%	0%	67
Standard Control	100%	0%	0%	22
5MXLCAB (15 ng ea)	89.8%	10.2%	0%	49
XLCA (10 ng)	64.3%	37.7%	0%	45
XLCA (20 ng)	18.2%	77.3%	4.5%	44
XLCB (10 ng)	54.8%	38.1%	7.1%	42
XLCB (20 ng)	7.3%	82.9%	9.8%	41
XLCAB (15 ng ea), vent^a^	86.7%	13.3%	0%	15
XLCAB (5 ng ea)	40%	60%	0%	20
XLCAB (10 ng ea)	44.4%	50%	5.6%	18
XLCAB (15 ng ea)	22.8%	48.6%	28.6%	70
**Splicing Blocking**				
Uninjected	100%	0%	0%	55
5MXLCSDA (15 ng)	68.9%	31.1%	0%	29
XLCSDA (15 ng)	0%	44.4%	55.6%	36
XLCSDB (15 ng)	33.3%	66.7%	0%	45
XLCSDAB (7.5 ng ea)	35.1%	64.9%	0%	37
XLCSDAB (15 ng ea)	13%	74%	13%	23
**Rescue Experiment**				
Uninjected	100%	0%	0%	32
XLCAB (15 ng ea)	25%	66.7%	8.3%	36
XLCAB (15 ng ea) + MR^b^ CRIM1-FL (200 pg)	71.4%	28.6%	0%	70

a : ventral blastmeres instead of dorsal blastmeres were injected with morpholino oligos.

b : MR means morpholino oligonucleotide resistant.

Since the absence of an eye served as a simple read-out for phenotype severity, we assessed changes from MOs injection in different amounts. There was a dose response for both translation and splicing blocking MOs and that each produced the same phenotype (Table 2). Injection of ventral blastomeres with the translation blocking XLCAB combination had a minimal effect (Table 2, vent). MOs (Table 1) in which 5 of the nucleotides were mismatched had a greatly reduced effect though this was not zero (Table 2). Since 5 nucleotide mismatch MOs are known to retain some activity at the concentrations used here [33] we also used the standard control MO (GeneTools) that has no measurable activity as a control and observed no obvious phenotype (Table 2).

We also determined whether co-injection of a MO-resistant *CRIM1* mRNA with the XLCAB MOs resulted in phenotypic rescue. Though we did not observe a complete reversal of the effects of the XLCAB MOs, the MO-resistant *xCRIM1* mRNA reduced the percentage of embryos showing small or missing eyes (Table 2). Together, the activity of both MO types in producing the same phenotype, the correlation of that phenotype with the expression domain of *CRIM1*, suppression of *CRIM1* mRNA splicing with XLCSDAB MOs and a degree of phenotype rescue with xCRIM1 expression suggest the antisense oligonucleotides are specific.

**Figure 2:**
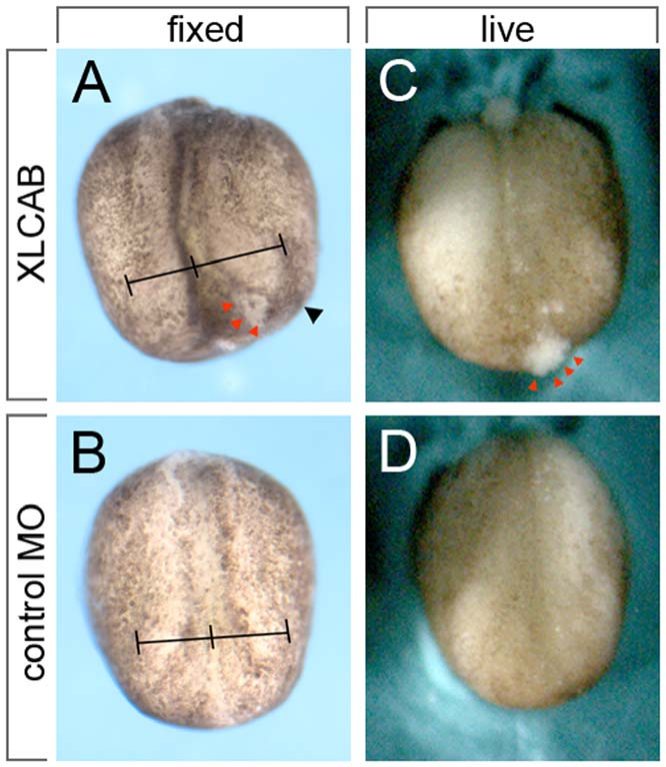
Disruption of neural fold morphogenesis in CRIM1 MO injected embryos. Pigmented embryos showed neural tube morphogenesis defects resulting from *CRIM1* MO injection. In (A and B) line intervals mark the distance from the midline to the neural folds to emphasize the defect in morphogenesis that is a result of *CRIM1* MO injection. In (A and C) red arrowheads indicate a region of cells that is sloughing from the embryo surface.

The absence of neural structures in tailbud stage embryos was consistent with the loss of neural plate integrity at earlier stages. Examination of XLCAB MOs injected pigmented embryos at stage 15 when neural plate morphogenesis is occurring revealed that the injected side had defects in neural plate formation. Specifically, failure of the neural plate boundary (the neural folds) to move toward the midline produced embryos with a pronounced asymmetry (Fig. 2). In many XLCAB MOs injected embryos, cells were seen sloughing from the surface of the injected side (Fig. 2, red arrowheads). In a typical experiment using 15 ng each XLCA and XLCB (Table 2), 20/70 embryos (28.6%) show severe cell sloughing. Time-lapse video microscopy of embryos bi-laterally injected with MOs (dorsal blastomeres, XLCAB at the 4-cell stage) in some cases showed a mild phenotype of delayed neural fold morphogenesis with a failure of anterior neural tube closure (Video S1) and in others a severe failure of cell adhesion across the entire neural plate (Video S2). This suggested that CRIM1 might have an essential role in promoting cell adhesion or suppressing cell death within the neural plate.

### Reduced cadherin junctional complexes is a primary consequence of CRIM1 loss-of-function

**Figure 3:**
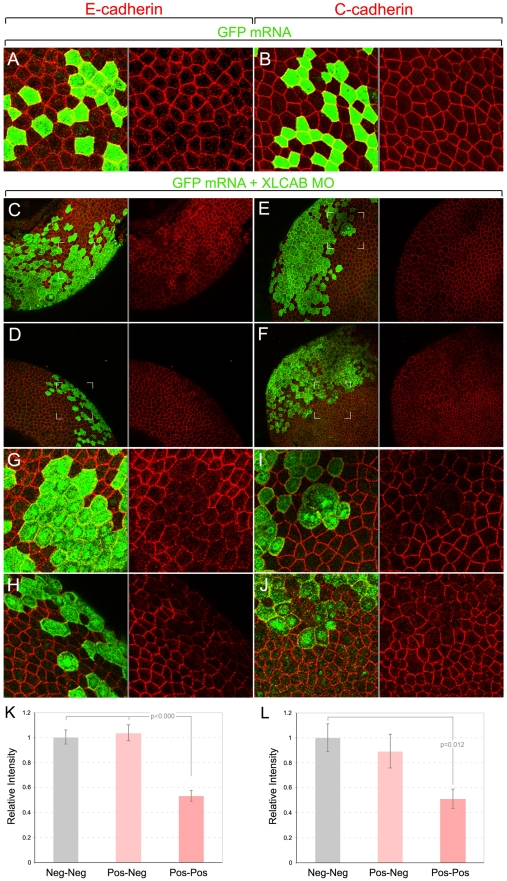
CRIM1 is required for junctional localization of E- and C-cadherin in the neural plate. (**A–J**) Immunofluorescence labeling of whole-mount *Xenopus* embryos after injection of translation-blocking XLCAB MOs. Embryos were co-injected with mRNA encoding GFP at the 4-cell stage and were fixed and labeled at stage 13 (early neurula) with antibodies to GFP (green), E-cadherin (A, C, D, G, H, red) or C-cadherin (B, E, F, I, J, red). Cadherin junctional complexes were visualized by combining multiple optical sections generated by confocal microscopy. In lower magnification images (C, D, E, F) it is apparent that tracer positive regions have lower levels of cadherin immunoreactivity and are irregularly shaped. In the magnified regions (G, H, I, J) indicated by white corner marks in (C, D, E, F) the loss of cadherin immunoreactivity in tracer positive cells is more obvious. The gray line between panels indicates separated color channels of the same image. (**K–L**) Graphs show the measured average E-cadherin (K) and C-cadherin (L) junctional staining intensity between two tracer-negative, one tracer negative and one tracer positive, or two tracer-positive cells (n = 20 pairs for each categories).

**Figure 4:**
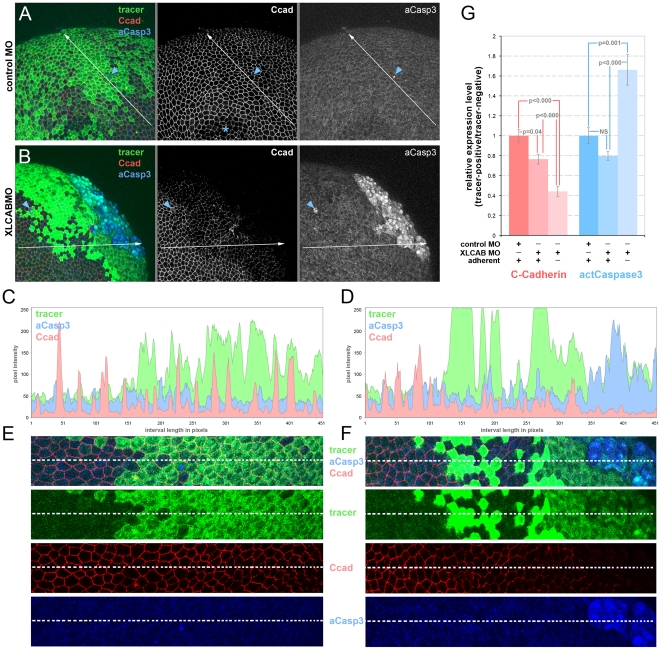
Cadherin junctional complex deficiency is the primary response to CRIM1 loss-of-function. (**A–B**) Embryos were injected with either standard control MO or 15 ng each XLCAB MOs with dextran tracer (green), fixed at stage 13 and labeled for C-cadherin (Ccad) and activated caspase 3 (aCasp3). In (A) the asterisk indicates a region where labeling in the apical junctional complex was not imaged due to the optical sectioning plane. In (A and B), the blue arrowheads indicate isolated dying cells labeled positive for activated caspase 3. (**C–D**) Three-channel histograms indicating pixel intensity along a line interval of 450 pixels in control MO (A and C) and XLCAB MO (B and D) injected embryos. (**E–F**) Magnified images of regions along the line interval in (A and B) with color channel merge (top) and separated color channels corresponding to different labels as indicated. (**G**) Quantification of relative expression levels of C-cadherin and activated Caspase 3 in standard control MO and XLCAB injected embryos. The expression levels are determined by the ratio of average pixel intensities over 150-pixel intervals in tracer-positive regions and tracer-negative regions within the same embryo. In XLCAB injected embryos, relative expression levels were measured over intervals placed exclusively in tracer-positive, adherent regions or tracer positive, non-adherent regions (n = 8 for all categories).

**Figure 5:**
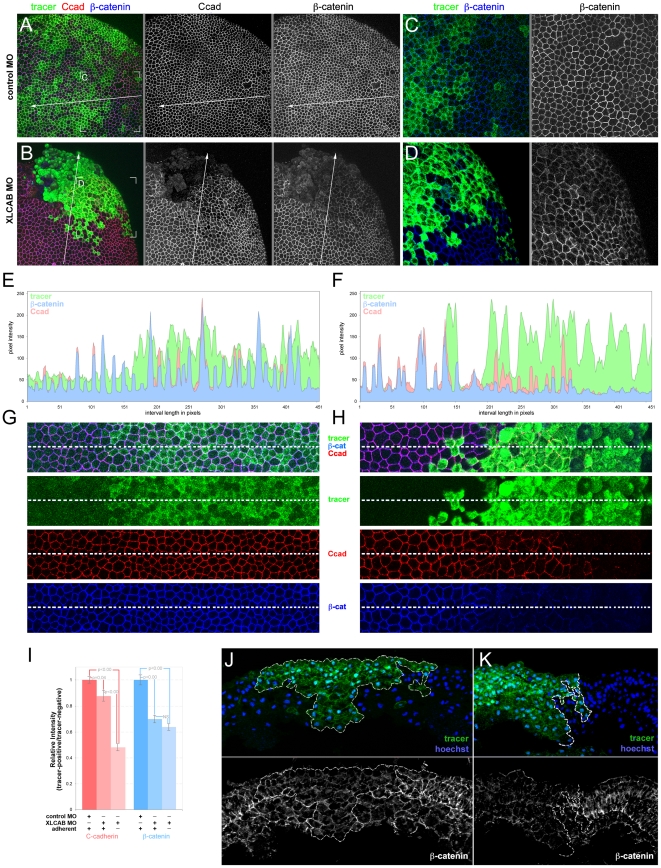
CRIM1 is required for junctional localization of ß-catenin in the neural plate. (**A–D**) Whole-mount immunofluorescence labeling of stage 13 *Xenopus* embryos in which standard control MO (A, C) or translation-blocking XLCAB MOs (B, D) were co-injected with dextran tracer at the 4-cell stage. Embryos were labeled with antibodies to C-cadherin and ß-catenin as indicated. (**C–D**) Higher magnifications of the indicated regions of (A and B). (**E–F**) Pixel intensity histograms of imuunofluorescence labeling for the line intervals shown in (A and B) respectively. (**G–H**) Magnified regions corresponding to the line intervals shown in (A and B) with color channel merge (top) and separated color channels corresponding to different labels as indicated. (**I**) Quantification of relative expression levels of C-cadherin and ß-catenin in standard control MO and XLCAB injected embryos by normalizing average pixel intensities over 150-pixel line intervals in tracer-positive regions to those of tracer-negative regions within the same embryo. In XLCAB injected embryos, relative expression levels were measured over intervals placed exclusively in tracer-positive, adherent regions or tracer positive, non-adherent regions (n = 20 in all categories) (J–K) Immunofluorescence labeling of cryosections from *Xenopus* embryos at stage 16 (mid-neurula) of mildly affected embryos. MOs were co-injected with dextran tracer (green). Cryosections were labeled with Hoechst 33258 for nuclei (blue) and with antibodies to ß-catenin (J, K, white). Tracer-positive regions are outlined with a dashed white line. The gray line between panels indicates separated color channels of the same image.

**Figure 6:**
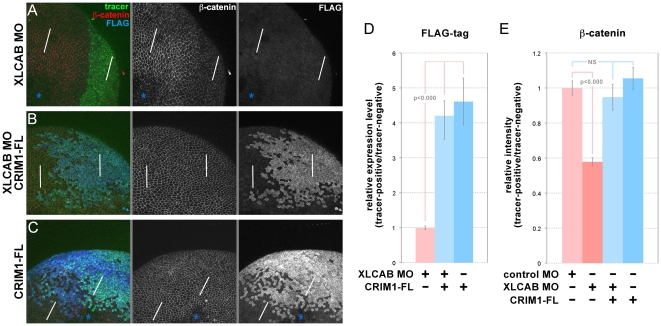
Restoration of CRIM1 expression can rescue ß-catenin level in CRIM1 loss-of-function embryos. (**A–C**) Whole-mount immunofluorescence labeling of stage 13 *Xenopus* embryos in which XLCAB MOs, MO-resistant mRNA of FLAG-tagged full-length CRIM1 (CRIM1-FL) or a combination of the two were injected at the 4-cell stage, together with dextran tracer. In (A and C) the asterisks indicate regions where labeling in the apical junctional complex were not imaged due to the optical sectioning plane. (**D**) Quantification of relative expression level of FLAG-tagged CRIM1 protein by calculating the ratio of average pixel intensity of FLAG labeling over 150 pixel line intervals placed in tracer-positive or tracer-negative regions (white bars in A–C). (**E**) Quantification of relative expression level of ß-catenin by calculating the ratio of average pixel intensity of ß-catenin labeling over 150 pixel line intervals placed in tracer-positive or tracer-negative regions (white bars in A–C).

To distinguish between these two possibilities, we first determined whether the level or localization of cadherins that are critical adhesion molecules in *Xenopus* neural plate [34] might be affected in *CRIM1* knockdown embryos. We coinjected XLCAB MOs with a tracer mRNA encoding GFP at the 4-cell stage and then performed whole-mount immunolabeling for cadherins at stage 13 (early neurula stage). In these preparations, an apical cadherin junctional complex is identified revealing patterns of cell packing and cell size at the surface (Fig. 3). In this case we controlled the experiment by injecting the GFP tracer mRNA alone. In other experiments co-injecting control MOs with dextran tracer gave identical results (Figs. 4, 5, 6). In control embryos we see slight junction-to-junction variation in labeling intensity for both E-cadherin (Fig. 3A) and C-cadherin (Fig. 3B), but this did not correlate with GFP expression.

By contrast, when the GFP mRNA and the *CRIM1* MO were co-injected there were dramatic changes in cadherin labeling in GFP expressing cells. At low magnification (Fig. 3C, D, E, F) tracer positive regions have reduced immunoreactivity for both E-cadherin and C-cadherin. Higher magnification (Fig. 3G, H, I, J) shows the precise correlation between GFP expression and reduced junctional labeling intensity. In addition, a junction between two tracer positive cells generally has a low level of cadherin immunoreactivity compared with junctions between a tracer positive and a tracer negative pair or between two tracer negative cell junctions (Fig. 3G, H, I, J). To quantify the E- and C-cadherin labeling, we measured pixel intensities over a curved line interval superimposed along junctional labeling between two cells. When normalized to the value of junctions between pairs of tracer-negative cells, a tracer positive-tracer negative pair showed no reduction in labeling intensity whereas tracer-positive pairs showed significantly reduced labeling intensity for E-cadherin (Fig. 3K) and C-cadherin (Fig. 3L). At higher magnification, some tracer positive cells have a rounded shape and a greater apical surface area than their tracer-negative neighbors (Fig. 3G, H, I, J) disrupting the pattern of cell packing.

While these changes in junctional cadherin levels and cell shape were consistent with a role for CRIM1 in adhesion, it remained possible that the cells with low cadherin levels were undergoing apoptosis as a primary response to CRIM1 loss-of-function. To determine whether this occurs, we performed two different assays for cell death. First, we injected embryos with either the fluorescent dextran tracer alone or with tracer plus 15 ng each XLCA and B MOs into a dorsal blastomere at the 4-cell stage. We harvested embryos at stage 13, permeablized and performed whole-mount TUNEL labeling (Fig. S1). As a positive control, we used the same combination of control and MO-injected embryos but treated them with DNase I to nick genomic DNA and enhance TUNEL labeling (Fig. S1). DNase I-treated embryos were TUNEL labeled; control or XLCAB-injected embryos without DNase-I treatment were not. Embryos were injected with the same amount of MOs that reliably caused reduced junctional cadherin labeling at the same analyzed stage (Fig. 3).

Since it can be argued that TUNEL labeling monitors a late event in the activation of cell death pathways, we also performed labeling for activated Caspase 3, an early marker of cell death pathway activation combined with labeling for C-cadherin (Fig. 4A and B). In this set of experiments, we analyzed CRIM1 knockdown embryos that showed a patch of de-adhering cells judged morphologically (Fig. 4B). We performed quantification of pixel intensity for the dextran tracer, C-cadherin and activated Caspase 3 along 450 pixel line intervals extending through tracer-negative to tracer-positive regions (Fig. 4A and B). These data are graphically represented in pixel intensity histograms (Fig. 4C and D). Regions of the micrograph containing the line interval are reproduced at higher magnification below the histogram (Fig. 4E and F). We analyzed 14 examples each of control MO and XLCAB-injected embryos and found consistent results.

In embryos co-injected with the tracer and the standard control MO, lineage tracer-positive cells retained strong C-cadherin junctional staining (Fig. 4A, middle panel and 4C, red). Activated caspase-3 levels, with the exception of the occasional positive cells (Fig. 4A, blue arrowhead), were consistently low across the whole embryo (Fig. 4A, right panel) and along the line interval used for analysis (Fig. 4C, blue). By contrast, in embryos co-injected with the tracer and 15 ng each XLCAB MO, C-cadherin labeling was consistently lower in tracer-positive regions as seen in the micrographs (Fig. 4B, middle panel, 4F, Ccad) and also when comparing red channel pixel intensities in tracer-negative and positive regions on the histogram (Fig. 4D).

We performed quantification of these signals by measuring pixel intensities over 150 pixel line intervals located exclusively in tracer negative (control MO and XLCAB injected embryos), tracer positive, adherent (control MO and XLCAB injected embryos), or tracer positive, non-adherent regions (XLCAB injected embryos only) in 8 different embryos. In XLCAB injected embryos, C-cadherin labeling was significantly reduced in both adherent and non-adherent tracer-positive regions compared with tracer-negative regions (Fig. 4G, red bars. A number lower than 1 indicates reduction of C-cadherin expression in MO injected regions). Importantly, adherent, MOs injected regions with reduced C-cadherin levels show no change in the level of activated Caspase 3 (Fig. 4G, blue bars). In addition, activated caspase 3 levels only increase dramatically when cells show non-adherent morphology (Fig. 4G, blue bars). These data argue that the primary consequence of CRIM1 loss-of-function is a diminished level of cadherin junctional complex and that cell de-adhesion followed by activation of cell death pathways is a secondary consequence.

### CRIM1 is required for ß-catenin localization to junctional complexes

The cadherin junction defects apparent in CRIM1 knockdown experiments prompted us to determine whether CRIM1 might regulate the level or distribution of other major adhesion complex proteins. To assess this, we generated embryos co-injected with the dextran tracer and control or XLCAB MOs and labeled for both C-cadherin and ß-catenin. As described above, we chose to analyze experimental embryos that had regions of non-adherent cells as judged morphologically (Fig. 5B). This analysis is illustrated and quantified as described for Fig. 4.

Control MO injected embryos showed levels of C-cadherin and ß-catenin signal that were consistent across tracer-negative and tracer-positive regions of the embryo (Fig. 5A, C, E, G). By contrast, tracer-positive regions in XLCAB-injected embryos showed reduced levels of both C-cadherin and ß-catenin regardless of whether these regions were adherent or non-adherent (Fig. 5B, D, F, H). To quantify the level of C-cadherin and ß-catenin, we generated pixel intensities over 150 pixel intervals on control MO-positive, and XLCAB-positive adherent and non-adherent regions. We then quantified the changes in average pixel intensities in MO-positive (tracer-positive) regions compare to those in MO-negative regions for both C-cadherin and ß-catenin labeling (Fig. 5I). Compared with control MO regions, the XLCAB MO resulted in a mild but statistically significant reduction in C-cadherin signal and a more pronounced reduction in ß-catenin signal (Fig. 5I). Interestingly, the level of C-cadherin signal reduced dramatically when cells become non-adherent while ß-catenin signal showed no further reduction (Fig. 5G, H, I). This suggested that a primary consequence of CRIM1 loss-of-function is the failure of ß-catenin to stably associate with cadherin junctional complexes.

Compromise of the cadherin junctional complex leads to defects in apical-basal epithelial polarity [5]. To determine if this feature of neural plate epithelial cells might be changed with CRIM1 loss-of-function, we performed similar experiments by co-injecting XLCAB with dextran tracer and performed immunofluorescent labeling on cross sections of the neural epithelium. Embryos displaying a mild phenotype were analyzed midway through neurulation at stage 16. The tracer was generally (Fig. 5K, green) but not always (Fig. 5J, green) distributed in a region that abutted the midline as would be expected for injection of a dorsal blastomere at the 4-cell stage. Tracer positive regions had a markedly different labeling pattern for ß-catenin. In unaffected neural epithelium (tracer negative, Fig. 5J, K) the neural epithelium has intense ß-catenin labeling at cell junctions and the columnar cell shape of the outermost epithelial layers is distinct (tracer negative, Fig. 5 J, K, grayscale panels). In all regions receiving the XLCAB MOs (Fig. 5 J, K, green region with dashed white line boundary) junctional ß-catenin labeling level is lower, the cells show a more rounded shape and the epithelium is disorganized. Out of 24 embryos each of experimental and control, we found polarity defects that were restricted to the tracer-positive regions in 7 experimental embryos.

We then determined whether restoration of CRIM1 expression would rescue the abnormal distribution of ß-catenin in CRIM1 knockdown cells. To this end, a MO-resistant, FLAG-tagged full-length *CRIM1* mRNA (CRIM1-FL) was co-injected with XLCAB MOs into a dorsal blastomere at the 4-cell stage. The expression level of the tagged protein was measured by comparing average pixel intensities over a 150 pixel line interval placed in tracer positive and tracer negative areas (Fig. 6 A, B, C, white lines). Injection of the mRNA resulted in robust expression of tagged full-length CRIM1 with or without co-injection of XLCAB MOs (Fig. 6B, C right panels, Fig. 6D blue bars). Whole-mount ß-catenin labeling was performed on embryos injected with different combinations of MOs and mRNA. We found that while injecting CRIM1 mRNA alone did not change the expression of ß-catenin (Fig. 6C, middle panel; Fig. 6E), co-injecting CRIM1 mRNA with XLCAB MOs restored the ß-catenin intensity (Fig. 6A, B middle panels) to the normal level of ß-catenin as in embryos injected with control MO (Fig. 6E). Combined, these data suggest CRIM1 has an essential role in stabilizing the cadherin junctions.

### CRIM1 complexes with ß-catenin and N-cadherin via its cytoplasmic domain

**Figure 7:**
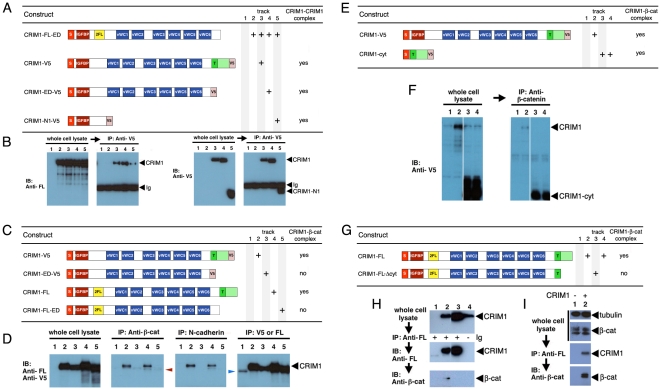
CRIM1 self-associates via the N-terminal domain and forms a complex with ß-catenin and N-cadherin via the C-terminal domain. (**A, C, E, G**) Tables showing CRIM1 expression constructs used in pull-down assays and result summaries. (**B**) According to anti-V5 immunoprecipitations from the same whole-cell lysates, CRIM1-V5 (track 3), CRIM1-ED-V5 (track 4) and CRIM1-N1-V5 (track 5) all interact with CRIM1-FL-ED suggesting multimerization via the N-terminal region represented in CRIM1-N1-V5. The immunoglobulin used for immunoprecipitation is detected in the immunoblot of (B), right panels of each pair (Ig). (**D**) Anti-V5 and anti-FLAG (FL) immunoblots of whole-cell lysates show all CRIM constructs shown in (C) express to abundant levels in 293 cells. Control anti-V5, anti-FLAG immunoprecipitation followed by anti-V5, anti-FLAG immunoblot shows that CRIM1 proteins are readily detected. Immunoblots of the same immunoprecipitations with anti-ß-catenin or anti-N-cadherin antibodies show that CRIM1 forms complexes with ß-catenin and N-cadherin but only if the CRIM1 cytoplasmic domain is present. The band in track 1 of D (blue arrowhead) is a background band. (**F**) Anti-V5 immunoblots of whole cell lysates (left panel) and anti-ß-catenin immunoprecipitations (right panel) show a version of CRIM1 with the signal peptide, trans-membrane domain and cytoplasmic domain forms a complex with ß-catenin. (H) immunoprecipitation using anti-FLAG antibodies in 293 cells expressing either CRIM1-FL or CRIM1-FLΔcyt. ß-catenin co-immunoprecipitates with CRIM1-FL, track 2, but not with CRIM1-FLΔcyt, track 3. Track 4 shows a control IP from 293 cells expressing CRIM1-FL where no primary antibody was added. No ß-catenin association is observed. (**I**) Co-immunoprecipitation of ß-catenin with FLAG-tagged CRIM1-FL with 293 whole cell lysate shows detection of endogenous ß-catenin. Anti-tubulin western blot was shown as loading control. In all experiments, an equal proportion of lysate is represented on compared gel tracks.

As a first step in understanding the mechanism of action of CRIM1, we determined whether multiple CRIM1 molecules could associate in a complex. We co-expressed a FLAG-tagged ectodomain form (Fig. 7A, top line) with a series of deletion mutants carrying C-terminal V5 tags (Fig. 7A) and determined whether this would coimmunoprecipitate (co-IP) from HEK293 cells. According to immunoblots with appropriate antibodies, all proteins expressed well (Fig. 7B, left panels) and the V5 tagged proteins could also be efficiently IPd (Fig. 7B, far right). Anti-V5 IP followed by immunoblot with anti-FLAG showed all deletion mutants of V5 tagged CRIM1 could form complexes with CRIM1-FL-ED (Fig. 7B, center left). These data indicate that CRIM1 can form complexes where multiple CRIM1 molecules are present. These data also show that an N-terminal region containing the IGFBP-like domain is sufficient for formation of this complex.

The apparent role of CRIM1 in stabilizing cadherin junctions shown by knockdown and rescue experiments prompted us to determine whether CRIM1 might directly interact with major adhesion complex proteins. To this end we over-expressed epitope-tagged CRIM1 in HEK293 cells, and determined whether CRIM1 could be IPd in these complexes (data not shown). When anti-ß-catenin antibodies were used for IP, CRIM1 was readily detected by immunoblot (data not shown). We then generated mutant forms of CRIM1 that lacked the cytoplasmic domain (Fig. 7C). We also used two different locations for epitope tagging given the possibility that a C-terminal epitope tag might prevent a cytoplasmic domain interaction (Fig. 7C). All four modified CRIM1 proteins expressed well in HEK293 cells (Fig. 7D, left panel) and could be IPd effectively with the antibody to the appropriate tag (Fig. 7D, right panel). Only CRIM1 with an intact cytoplasmic domain would form a complex with ß-catenin (Fig. 7D, center left) through IP using anti- ß-catenin antibodies.

To determine whether the cytoplasmic domain of CRIM1 was sufficient for ß-catenin complex formation, we expressed CRIM1-cyt (consisting of the secretory leader, transmembrane and cytoplasmic domains, Fig. 7E) in 293 cells and performed ß-catenin IPs. Both CRIM1-cyt and the full-length CRIM1 expressed well as indicated by an anti-V5 immunoblot of cell lysates (Fig. 7F, left panel – tracks 3 and 4 are duplicates). Using anti-ß-catenin antibodies, both full-length CRIM1 and CRIM1-cyt co-IPd (Fig. 7F, right panel). We used antibodies to the FLAG epitope in CRIM1-FL and CRIM1-FLΔcyt (Fig. 7G) in reciprocal IPs and detected ß-catenin (Fig. 7H) in immunoblots. In lysates from CRIM1-FL expressing cells, total ß-catenin levels appeared unchanged where a CRIM1-ß-catenin complex was demonstrated via co-IP (Fig. 7I). These data provide strong evidence that CRIM1 and ß-catenin exist in the same complex. We could not convincingly demonstrate a direct interaction between a variety of recombinant forms of the CRIM1 cytoplasmic domain and ß-catenin *in vitro* (data not shown).

The CRIM1 knockdown adhesion defect, together with co-existence of CRIM1 and ß-catenin in a protein complex raised the possibility of CRIM1 association with cadherins. N-cadherin is expressed in HEK293 cells whereas E-cadherin is not (data not shown). When CRIM1 was over-expressed, anti-N-cadherin antibodies IPd CRIM1 (Fig. 7D). Formation of a CRIM1-N-cadherin complex was also dependent upon the presence of an intact CRIM1 cytoplasmic domain (Fig. 7C, D). Combined, these data indicate that CRIM1 can form complexes with ß-catenin and N-cadherin via its cytoplasmic domain. This, with reduced junctional cadherin levels in *Xenopus CRIM1* knockdown expreriments, suggested that the adhesion defect resulted from disruption of cadherin-dependent junctional complexes.

## Discussion

In this report we assessed the function and mechanism of action of the unique transmembrane molecule cysteine-rich motor neuron 1 (CRIM1). Using antisense oligonucleotide knockdown experiments in *Xenopus laevis*, we showed that CRIM1 is essential for formation of the nervous system. Since the expression of early neural markers is unaffected, CRIM1 clearly did not regulate the inductive phases of neural development when BMP signaling is involved. Rather, we provide evidence at both the cellular and protein levels that CRIM1 is required for formation of cadherin-dependent adhesion junctions. Specifically we show that CRIM1 can form complexes with ß-catenin and cadherins and that these proteins are reduced in junctional complexes of CRIM1 knockdown *Xenopus* embryos. Combined, these data suggest normally, CRIM1 is critical for the formation of cadherin junctions in the developing neural plate. These findings raise several questions.

### CRIM1 function in cadherin-mediated morphogenesis

The classical cadherins have diverse roles in development and homeostasis including mechanical cell-cell adhesion, coordination of cell movements during morphogenesis, establishment and maintenance of epithelial polarity as well as cell-to-cell signaling and recognition [35]. There are different ways in which these various cadherin activities are regulated, some are post-transcriptional and therefore mediated by the interaction of cadherins with other proteins. The association of ß-catenin with cadherins is regulated by different phosphorylation states that have either positive (serine phosphorylation of E-cadherin or ß-catenin) or negative (tyrosine phosphorylation of ß-catenin) effects on complex formation [36]. Other levels of cadherin negative regulation include cleavage of the extracellular domain by ADAM (a disintegrin and metalloprotease domain) 10 [37] and cleavage of the intracellular domain by proteases such as γ-secretase/presenilin-1 [38] thus promoting disassembly of the cadherin complex. Cadherin endocytosis into clathrin-coated vesicles [39] may also negatively regulate cell-cell junctional adhesiveness perhaps as a consequence of the loss of p120 catenin association [40].

In this study, we show that CRIM1 has an essential role in cell-cell adhesion during development of the central nervous system. CRIM1 appears to lack any intrinsic capacity to mediate cell-cell adhesion (unpublished results) yet it seems essential for the formation or stabilization of cadherin-dependent adhesion complexes. A comparison of the expression patterns of CRIM1 and cadherins within epithelia reveals that CRIM1 is expressed in sub-regions within larger cadherin-positive domains. An example is the presumptive lens in the mouse where CRIM1 is first expressed in a patch of ectoderm that will invaginate to form the lens pit [19]. This small region of presumptive lens ectoderm is part of the larger embryonic head ectoderm that expresses E-cadherin [41]. Similarly, the region of the *Xenopus* neural plate that expresses CRIM1 is part of a larger surface ectoderm that expresses cadherins [42], [43], [44]. The CRIM1 expressing neural plate will, like the presumptive lens, undergo dramatic morphogenesis at the time CRIM1 is expressed [45]. The mild morphogenesis phenotype observed in the CRIM1 knockdown experiments is similar to the failure of hinge point formation in neural tube bending induced by knockdown of the actin associated protein, Shroom [46]. Connectivitity to the cytoskeleton is important for stabilization of cadherin junctional complexes. CRIM1 is not obligatorily expressed in cadherin-positive regions suggesting that it is not universally required for the formation or stabilization of adhesion complexes. CRIM1 may become essential for the function of cadherin where it is expressed, perhaps displacing another cadherin complex stabilization mechanism thus regulating adhesive activity perhaps during morphogenesis.

Cell-cell adhesion between animal cells undergoing normal morphogenetic movements, as in the bending of epithelial sheets, must be dynamic without losing cell-cell contact. Kametani and Takeichi demonstrated basal-to-apical cadherin flow occurs at cell junctions between moving transformed cells in culture [47]. They visualized junctional instability and cadherin-catenin-actin protein rearrangements at sites of cellular morphogenesis while maintaining cell contact. CRIM1 may play role in regulating cadherin-catenin junctional stability. We show CRIM1 interaction with these proteins and expression in sites where epithelial sheet bending and dynamic cellular rearrangement occurs.

### CRIM1 mechanism of action

Beyond the demonstration that the 82 residue intracellular domain of CRIM1 is required for association with ß-catenin and cadherins, the mechanism of complex formation is unclear. The cytoplasmic domain of CRIM1 is highly conserved but does not have obvious interaction motifs. In particular, there are no primary sequence features of typical ß-catenin ligands. Proteins that bind ß-catenin in an extended conformation along the armadillo repeat (ARM) domain (such as the cadherins, ICAT, TCFs, APC) are characterized by a DXΘΘXΦX_2–7_E motif where Θ is an aliphatic residue, and Φ an aromatic residue [48]; there is no such motif in CRIM1. Furthermore, ligands that bind in the positively charged groove of the ß-catenin ARM domain are typically acidic (calculated pI (isoelectric point) for the cadherins is 3.3, for APC, 4.1, and for the Tcf family, 4.4). The calculated pI of the CRIM1-cytoplasmic domain is 9.8.

Thus, it may not be surprising that we were unable to demonstrate a direct interaction of recombinant forms of the CRIM1 cytoplasmic domain and ß-catenin or between the CRIM1 cytoplasmic domain and the N-cadherin cytoplasmic domain (data not shown). This suggests that the formation of a complex between CRIM1, ß-catenin and cadherin may depend on additional proteins that might have a bridging activity or perhaps on post-translational modifications.

Association of ß-catenin with cadherins in the endoplasmic reticulum (ER) is important for efficient transit of the complex to the plasma membrane and formation of adhesion complexes [49]. Some characteristics of CRIM1 are consistent with participation in this pathway. For example, in vascular endothelial cells, CRIM1 moves to the membrane from the ER upon activation with an inflammatory stimulus [21]. It has also been shown that that CRIM1 can interact with bone morphogenetic proteins via its extracellular domain and can retain them in the ER as a way of suppressing their activity [22]. Combined with data presented in this report, these findings might suggest that a critical cellular location for CRIM1 is the ER and furthermore, that CRIM1 might associate with ß-catenin and cadherins in this location. Further investigation of this proposal is required.

## Supporting Information

Figure S1 **Cell apoptosis does not occur prior to loss of junctional cadherions in CRIM1 MO injected embryos.** **(A and B)** TUNEL labeling of stage 13 control (A) or XLCAB injected (B) embryos with color channel merge on top and TUNEL channel alone at bottom. **(C and D)** TUNEL labeling of stage 13 control (C) or XLCAB injected (D) embryos treated with DNase I to manually nick genomic DNA. Color channel merges are on top and TUNEL alone shown at bottom.
(TIF)

Video S1 (MPEG)

Video S2 (MPEG)
