# Supplementary figures and images for: CRIM1 Complexes with ß-catenin and Cadherins, Stabilizes Cell-Cell Junctions and Is Critical for Neural Morphogenesis
Source: PLoS One. 2012 Mar 12;7(3):e32635. doi: 10.1371/journal.pone.0032635 (PMC3299674; doi:10.1371/journal.pone.0032635)

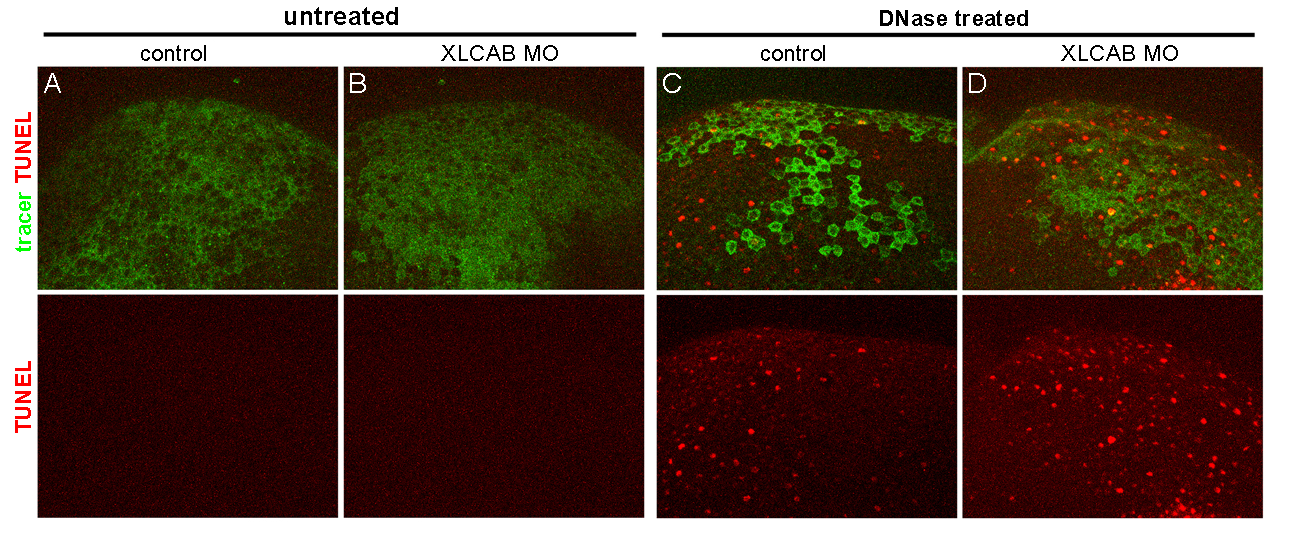

Supplement: Figure S1 — Cell apoptosis does not occur prior to loss of junctional cadherions in CRIM1 MO injected embryos. (A and B) TUNEL labeling of stage 13 control (A) or XLCAB injected (B) embryos with color channel merge on top and TUNEL channel alone at bottom. (C and D) TUNEL labeling of stage 13 control (C) or XLCAB injected (D) embryos treated with DNase I to manually nick genomic DNA. Color channel merges are on top and TUNEL alone shown at bottom. (TIF) [file pone.0032635.s001.tif]
